# Artificial intelligence-enabled opportunistic identification of immune checkpoint inhibitor-related adverse events using [^18^F]FDG PET/CT

**DOI:** 10.1007/s00259-025-07364-2

**Published:** 2025-05-29

**Authors:** Clemens P. Spielvogel, Aleksa Lazarevic, Lucia Zisser, David Haberl, Christophoros Eseroglou, Lucian Beer, Marcus Hacker, Raffaella Calabretta

**Affiliations:** 1 Division of Nuclear Medicine, Department of Biomedical Imaging and Image-guided Therapy, Medical University of Vienna, Spitalgasse 23, Vienna, A-1090 Austria; 2 Division of General and Pediatric Radiology, Department of Biomedical Imaging and Image-Guided Therapy, Medical University of Vienna, Vienna, Austria

**Keywords:** Immune checkpoint inhibitors, Adverse events, FDG, Artificial intelligence

## Abstract

**Purpose:**

Immune checkpoint inhibitors (ICI) have transformed cancer therapy, improving outcomes in malignancies like lung cancer, melanoma, and lymphoma by targeting PD-1, PD-L1, and CTLA-4 to enhance T cell-mediated tumor destruction. However, ICI often induce immune-related adverse events (irAEs) across multiple organs. [^18^F]FDG PET/CT is a valuable tool for assessing immune activation, but whole-organ inflammation evaluation remains time-consuming and prone to variability. This study investigates AI-driven organ-level [^18^F]FDG PET/CT uptake changes pre- and post-ICI therapy to opportunistically detect irAEs.

**Methods:**

A total of 64 patients with lung cancer, melanoma, or lymphoma who underwent [^18^F]FDG PET/CT before and after ICI therapy were included. Automated delineation of anatomical structures was performed using an artificial intelligence approach involving multiple subsequently applied deep learning models. SUV_max_ and SUV_mean_ were quantified for irAE-related organs (thyroid, myocardium, pancreas, liver, bone, spleen, and adrenal glands) after automated tumor lesion removal. Statistical analysis identified uptake changes linked to clinically observed inflammation-related adverse events.

**Results:**

Among 64 patients (mean scan interval 4.5 months), thyroid uptake increased post-ICI (ΔSUV_max_=0.44, *p* = 0.04; ΔSUV_mean_=0.22, *p* = 0.08). Increased uptake occurred in 55% (SUV_max_) and 53% (SUV_mean_) of patients. Thyroid-related adverse events were more frequent in those with increased uptake (29% vs. 3%, *p* = 0.008), with uptake increase being the most pronounced in the cases of hypothyroidism (*p* = 0.002 for SUV_max_, *p* = 0.03 for SUV_mean_). Myocardial, pancreatic, and hepatic uptake increased but without statistical significance (*p* > 0.05).

**Conclusion:**

AI-enabled opportunistic [^18^F]FDG PET/CT screening effectively detects thyroid-related irAEs, particularly hypothyroidism, through uptake quantification. This approach offers a promising biomarker for early irAE detection, enhancing patient management and immunotherapy optimization.

**Graphical Abstract:**

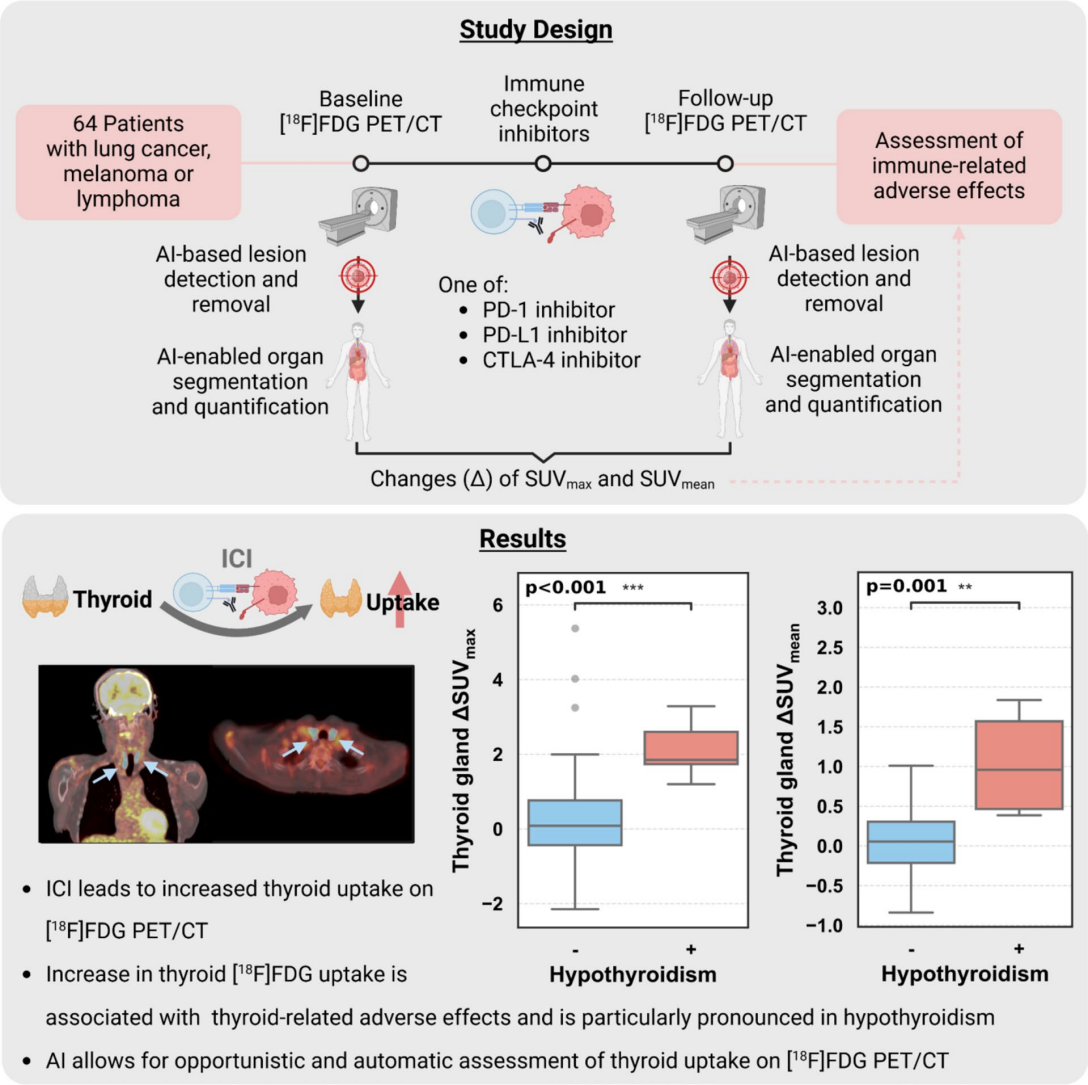

**Supplementary Information:**

The online version contains supplementary material available at 10.1007/s00259-025-07364-2.

## Introduction

Immune checkpoint inhibitors (ICI) have transformed cancer treatment, leading to significantly improved clinical outcomes across a range of malignancies by blocking immune checkpoint pathways, such as programmed cell death protein-1 (PD-1), its ligand PD-L1, and cytotoxic T-lymphocyte-associated protein 4 (CTLA-4), enabling cytotoxic T cells to recognize and attack cancer cells more effectively [1]. While effective, ICIs can cause immune-related adverse events (irAEs) affecting multiple organ systems through off-target immune activation [2, 3].

Due to its sensitivity to inflammation-related glucose metabolism, recent studies highlight the utility of 2-[^18^F]fluorodeoxyglucose ([^18^F]FDG) positron emission tomography/computed tomography (PET/CT), routinely performed for clinical purposes, in quantifying immune activation and inflammatory activity in different organs [4, 5]. Elevated organ uptake such as in the colon, thyroid, and lungs has been associated with irAEs [6, 7]. Thyroid dysfunction, particularly hypothyroidism, is among the most common endocrine irAEs and reflects the thyroid’s vulnerability to immune-mediated damage [8, 9]. However, distinguishing pathological immune activation from physiological uptake remains challenging. Mechanistically, irAEs arise from ICI-induced T cell hyperactivation and loss of immune tolerance, leading to off-target tissue inflammation. These immune responses may be detectable on [^18^F]FDG PET before symptoms appear, but the exact relationship between immune activation and imaging patterns is not fully understood.

Recently, artificial intelligence (AI)-based organ and lesion segmentation on [^18^F]FDG PET have made significant strides, with studies steadily moving beyond technical validation towards clinical validation and application [10, 11].

This study investigates the relationship between ICI therapy, [^18^F]FDG PET uptake, and irAEs on the organ-level. By analyzing changes in [^18^F]FDG uptake pre- and post-ICI treatment using an opportunistic quantification approach enabled by AI, we aim to better understand the role of immune activation in mediating adverse events and explore its potential implications for patient management.

## Materials and methods

### Study design and participants

Patients with either lung cancer, melanoma, or lymphoma who underwent [^18^F]FDG PET/CT scans before and after immunotherapy with ICI at the Division of Nuclear Medicine (Medical University of Vienna) were retrospectively included in this study. Patients had previously been included in other studies [12–14].

For both, the pre- and post-therapeutic PET/CT scans, a deep learning-based lesion segmentation was performed to subtract tumor lesions from the delineations of target organs. Subsequently, whole-body organ segmentation was performed based on a foundational deep learning model and organ-level SUV_max_ and SUV_mean_ were quantified. Differences (Δ) between pre- and post-ICI organ [^18^F]FDG uptake were assessed and associated with irAEs. At the time of pre- and post-therapeutic scans, patients were not under treatment with anti-inflammatory drugs. Included patients did not have concurrent inflammatory conditions. The study was conducted in alignment with the Declaration of Helsinki, and approved by the institutional review board of the Medical University of Vienna (1367/2020).

Included irAEs are defined as any clinically diagnosed inflammatory reaction to the ICI treatment, in addition to its therapeutic effects. We considered a composite of “thyroid-related adverse events” as well as thyroiditis, hypothyroidism, hyperthyroidism, hepatitis, and colitis. The prevalence of each considered irAE is shown the Table 1. A detailed description of thyroid-related adverse events is shown in the Supplement.

**Table 1:** Clinical characteristics

Parameter	Parameter value	*p* value	Entire data	Hypothyroidism neg.	Hypothyroidism pos.
Number of patients, n (%)			64 (100.00%)	58 (90.62%)	6 (9.38%)
Age (years)		0.9389	68.0 (14.4)	67.9 (14.6)	69.0 (12.1)
Sex					
	Male	0.6810	35 (54.69%)	31 (53.45%)	4 (66.67%)
	Female		29 (45.31%)	27 (46.55%)	2 (33.33%)
Cancer type					
	Lung cancer	0.3428	36 (56.25%)	31 (53.45%)	5 (83.33%)
	Lymphoma		8 (12.5%)	8 (13.79%)	0 (0%)
	Melanoma		20 (31.25%)	19 (32.76%)	1 (16.67%)
ICI drugs					
	Atezolizumab	0.5281	9 (14.06%)	7 (12.07%)	2 (33.33%)
	Durvalumab		3 (4.69%)	3 (5.17%)	0 (0%)
	Ipilimumab		1 (1.56%)	1 (1.72%)	0 (0%)
	Ipilimumab + Nivolumab		1 (1.56%)	1 (1.72%)	0 (0%)
	Nivolumab		16 (25.0%)	16 (27.59%)	0 (0%)
	Pembrolizumab		34 (53.12%)	30 (51.72%)	4 (66.67%)
ICI molecules					
	CTLA-4 + PD-1 inhibitors	0.8397	1 (1.56%)	1 (1.72%)	0 (0%)
	CTLA-4 inhibitor		1 (1.56%)	1 (1.72%)	0 (0%)
	PD-1 inhibitor		50 (78.12%)	46 (79.31%)	4 (66.67%)
	PD-L1 inhibitor		11 (17.19%)	9 (15.52%)	2 (33.33%)
	PD-L1-inhibitor		1 (1.56%)	1 (1.72%)	0 (0%)
Adverse events					
	None	0.0732	38 (59.38%)	32 (55.17%)	6 (100.0%)
	Thyroid-related side effects	< 0.0001	11 (17.19%)	5 (8.62%)	6 (100.0%)
	Hepatitis	1.0000	3 (4.69%)	3 (5.17%)	0 (0%)
	Colitis	1.0000	4 (6.25%)	4 (6.9%)	0 (0%)
	Thyroiditis	1.0000	1 (1.56%)	1 (1.72%)	0 (0%)
	Hyperthyroidism	1.0000	4 (6.25%)	4 (6.9%)	0 (0%)
	Hypothyroidism	< 0.0001	6 (9.38%)	0 (0%)	6 (100.0%)
Thyroid PET/CT marker					
	Post-treatment SUV_max_	0.0001	3.49 (1.58)	3.23 (1.38)	5.89 (1.34)
	Pre-treatment SUV _max_	0.2276	3.06 (1.09)	2.98 (1.02)	3.77 (1.46)
	Post-treatment SUV_mean_	< 0.0001	2.06 (0.85)	1.91 (0.72)	3.49 (0.63)
	Pre-treatment SUV_mean_	0.0495	1.85 (0.65)	1.78 (0.57)	2.45 (0.92)
	ΔSUV_max_	0.0003	0.44 (1.41)	0.26 (1.35)	2.12 (0.71)
	ΔSUV_mean_	0.0013	0.22 (0.7)	0.13 (0.65)	1.04 (0.59)

### Acquisition of imaging and clinical data

Examinations were performed using the same routine imaging protocol with a 64-slice multi-detector-row hybrid Biograph TruePoint 64 PET/CT device with Iomeron contrast enhancement. Demographic and blood parameters were acquired from the local hospital information system.

### Delineation and organ quantification

To ensure high accuracy, robustness to inter-rater variability, and to allow for speed making clinical application feasible, we performed any delineations fully automatically. An initial CT segmentation was facilitated by the application of TotalSegmentator, a foundational deep learning segmentation model, which is based on the nnUnet framework, trained on more than 1,200 CT images and which has been extensively validated in multiple studies [15, 16]. The resulting CT-based segmentation masks were subsequently transferred to the co-registered PET modality to define organ regions on PET. To account for inaccuracies of the segmentation and compensate for any potential co-registration artifacts, segmentation masks of the organs were eroded by 4 mm. To account for tumor-related uptake within the target organs, a deep learning lesion segmentation model based on a nnU-Net trained on the AutoPET dataset was applied [17, 18]. The resulting lesion delineations were visually confirmed. If a lesion was located in a target organ, the lesion was removed from the organ’s delineation. The resulting PET delineations were visually assessed to ensure correctness and subsequently quantified based on the entire delineated organ after erosion and lesion removal. Both, [^18^F]FDG SUV_max_ and [^18^F]FDG SUV_mean_ were quantified for the thyroid, myocardium, pancreas, liver, bone, spleen, and adrenal glands. Additionally, we assessed the spleen-to-liver ratio based on the SUV_mean_ of the corresponding spleen and liver delineations.

### Statistical analysis

For group comparisons, a t-test was performed if a Shapiro-Wilks test indicated normality and a Mann-Whitney U test for independent samples otherwise. For comparisons of the same patients before and after treatment, a paired t-test was performed for normally distributed data and a Wilcoxon signed-rank test for non-normally distributed data. For categorical variables, we used either a Fisher’s exact test or Chi-square test. *P* values are to be interpreted exploratorily. All analyses were performed using Python 3.9.5 and SciPy [19].

## Results

### Patient characteristics

Sixty-four patients (mean age 68.0 ± 14.4, 29 (45%) females) with histologically-proven lung cancer (*n* = 36), melanoma (*n* = 20), or lymphoma (*n* = 8) who underwent [^18^F]FDG PET/CT scans before and after immunotherapy with ICI (time interval between pre- and post-scans: 4.5 ± 4.5 months) were retrospectively included in this study. Clinical characteristics are displayed in Table 1.

### Changes after ICI treatment

**Fig. 1:**
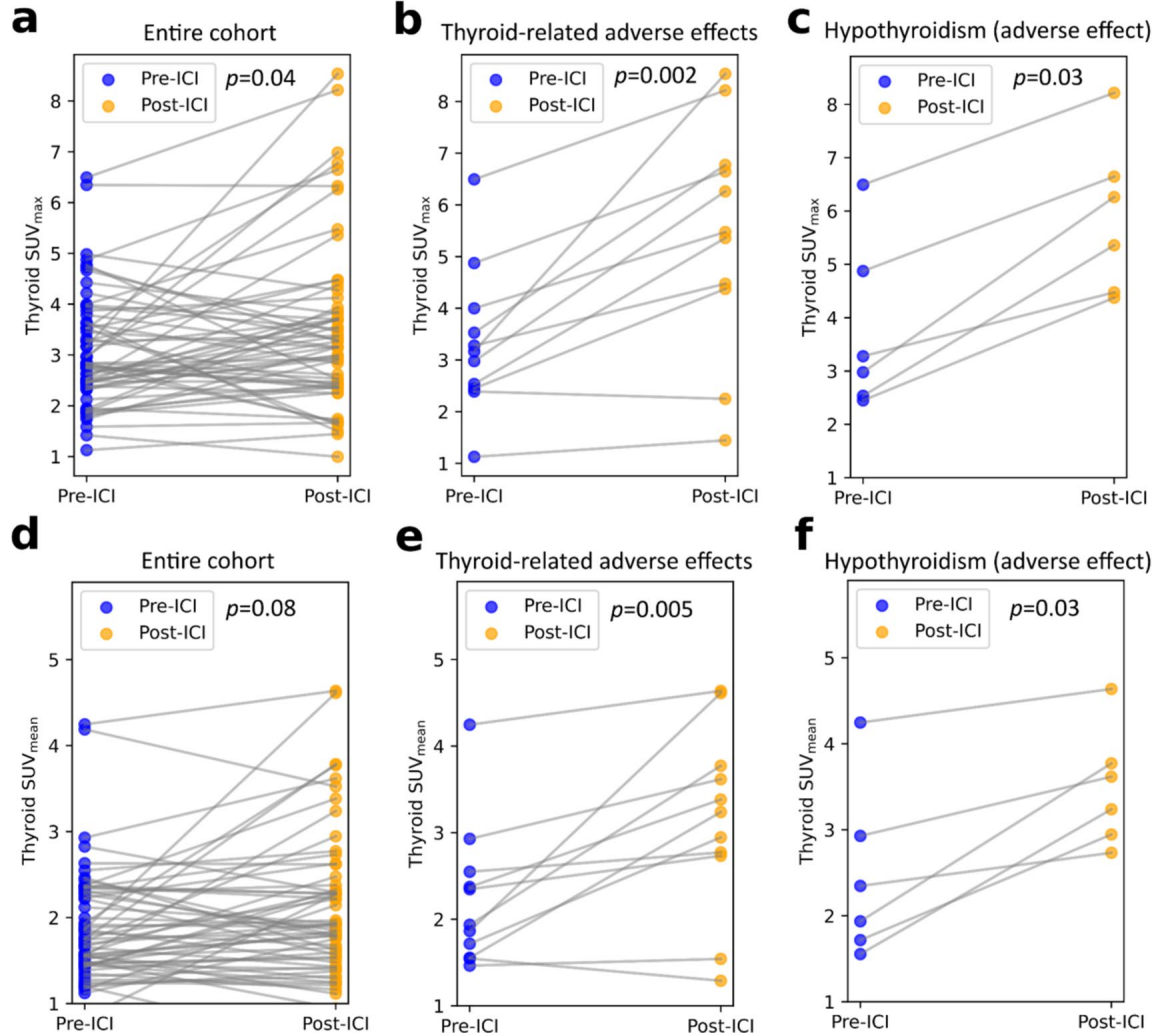
Thyroid SUV_max_ (**a**-**c**) and SUV_mean_ (**d**-**f**) before and after treatment for the entire cohort (**a**, **d**), thyroid-related adverse effects (**b**, **e**), and patients with hypothyroidism (**c**, **f**)

**Fig. 2:**
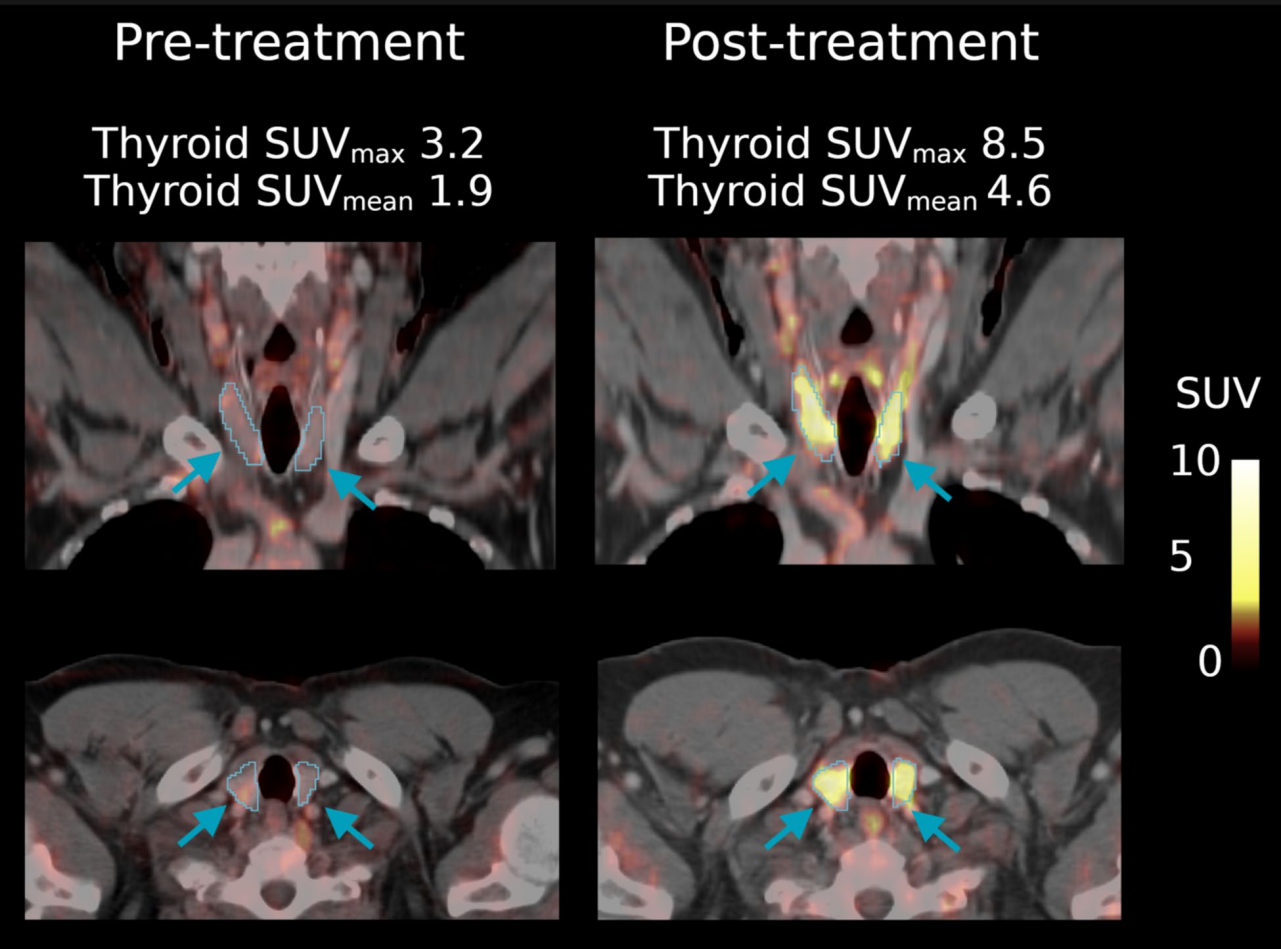
Example patient before (left) and after (right) treatment. The upper row shows a coronal slice and the lower row a transversal slice of the same patient. AI-based thyroid delineations are outlined in light blue

Patients had an increased thyroid [^18^F]FDG metabolic activity, which was significant when measured as SUV_max_ (*p* = 0.04), however non-significant when measured as SUV_mean_ (*p* = 0.08), after versus before ICI treatment (Fig. 1). On average, fT4 levels were reduced by 0.12 ± 0.59 ng/dL (*n* = 27) and fT3 was reduced by a mean of 0.81 ± 1.31 pg/mL (*n* = 21) before versus after therapy, however, not significantly (*p* = 0.16 and 0.18 respectively). Only 1/11 (9%) patients with thyroid-related adverse effects received longitudinal fT3 and fT4 tests (fT3 and fT4 reduction by 1.23 and 1.66 pg/mL respectively). Other organs including heart (SUV_max_ median pre- 7.72 vs. post-treatment 7.83, *p* = 0.61; SUV_mean_ 2.37 vs. 2.48, *p* = 0.99), pancreas (SUV_max_ 3.41 vs. 3.48, *p* = 0.40; SUV_mean_ 1.58 vs. 1.60, *p* = 0.53), liver (SUV_max_ 5.17 vs. 5.24, *p* = 0.79; SUV_mean_ 2.33 vs. 2.31, *p* = 0.48), hip bone (SUV_max_ 3.86 vs. 3.75, *p* = 0.49; SUV_mean_ 1.17 vs. 1.18, *p* = 0.85), and adrenal glands (SUV_max_ 2.50 vs. 2.69, *p* = 0.43; SUV_mean_ 1.78 vs. 1.70, *p* = 0.21) did not show changes in [^18^F]FDG uptake after immunotherapy. An example patient with increased thyroid uptake after ICI treatment is shown in Fig. 2 with uptake in other organs shown in Supplemental Fig. 1. A comparison of pre- versus post-ICI treatment uptake for target organs apart from the thyroid is shown in Supplemental Fig. 2.

### Association of uptake with adverse events

**Fig. 3:**
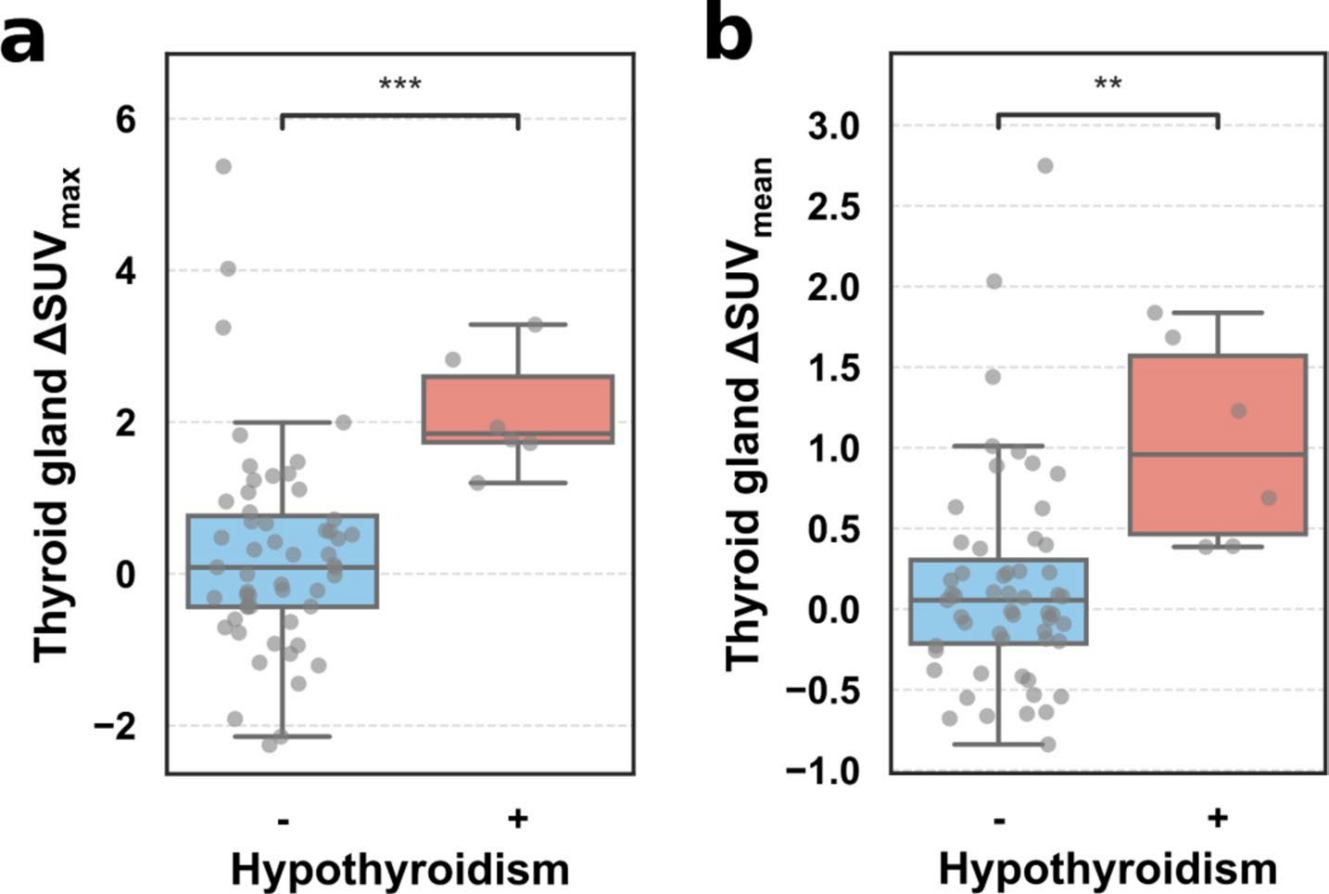
Changes in SUV_max_ (**a**) and SUV_mean_ (**b**) of patients with (+) and without (-) hypothyroidism

Patients with increased thyroid uptake (above the median ΔSUV_max_) were significantly more commonly affected by thyroid-related adverse events during treatment (1 versus 10 patients, odds ratio = 13.8, *p* = 0.006). Patients with thyroid-related adverse events after therapy had a mean increase in SUV_max_ after versus before therapy of 2.09 ± 1.54 while patients without thyroid-related uptake only had a mean increase in SUV_max_ of 0.08 ± 1.12 (*p* < 0.001). The results remained robust for SUV_mean_ with a mean increase of 0.91 ± 0.90 for patients with side effects and a mean increase of 0.07 ± 0.56 for patients without any thyroid-related adverse events (*p* = 0.002). Among patients with thyroid-related adverse events 6/11 (55%) had hypothyroidism. Patients with hypothyroidism alone had a significantly increased thyroid uptake in comparison to all other patients (mean ΔSUV_max_ 0.26 vs. 2.12, *p* < 0.001 and mean ΔSUV_mean_ 0.13 vs. 1.04, *p* = 0.001) (Fig. 3). Thyroid-related side effects occurred most commonly in lung cancer patients with 10/11 (91%) patients affected by lung cancer and the remaining patient being associated with melanoma. In total, 6/11 (55%) patients were treated with the PD-1 inhibitors pembrolizumab or nivolumab while the remaining 5 patients were treated with the PD-L1 inhibitors atezolizumab or durvalumab. Among the 6 patients associated with hypothyroidism, 2 received atezolizumab and 4 received pembrolizumab. Hyperthyroidism was less common than hypothyroidism with 4/11 (36%) cases. The remaining patient (1/11, 9%) was affected by autoimmune thyroiditis. Among the other organs, only ΔSUV_max_ of the spleen (*p* = 0.02) and ΔSUV_mean_ of the pancreas were significantly associated with irAEs (Supplemental Fig. 3).

## Discussion

ICI have significantly improved cancer treatment, even in cases with poor prognosis. This is particularly true for melanoma, lung cancer, and renal cell carcinoma [20, 21] Nevertheless, their non-specific immune activation frequently leads to irAEs, which differ from traditional treatment side effects and primarily affect the endocrine, gastrointestinal, skin, and cardiovascular systems [22, 23]. Due to their often vague symptoms, timely diagnosis and management require heightened clinical awareness [24, 25].

Studies suggest that certain irAEs, even those without clear clinical symptoms, may be identifiable using PET imaging [26]. Inspired by this, we investigated whether AI-enabled organ delineation and quantification of [^18^F]FDG PET/CT images could opportunistically detect irAEs by measuring organ-specific metabolic changes in a fast, accurate, and standardized fashion. The ability of AI to identify subtle image patterns supports its growing role in diagnostic imaging, with opportunistic screening having been proposed as a particularly intriguing concept [27, 28].

Our findings indicate that approximately half of the patients exhibited increased thyroid [^18^F]FDG uptake after ICI therapy, correlating with a higher incidence of thyroid-related irAEs, particularly hypothyroidism. This suggests that AI-driven, automated PET quantification may enable early irAE detection and prediction. Conversely, metabolic activity in other organs such as the myocardium, pancreas, liver, bone, spleen, and adrenal glands showed no significant post-treatment changes, supporting their lack of irAE involvement. While we expect the AI-assisted assessment to be accurate, its main advantage is not necessarily the increased diagnostic accuracy. When it comes to segmentation-based tasks, AI approaches excel in their ability to entirely remove inter-reader variability, leading to results that can be compared over multiple centers and studies. Further, while accurate, manual segmentation by imaging experts not only exhibits inter-reader variability but is also cumbersome and time-consuming. AI-based approaches on the other hand can accomplish the segmentation of multiple organs within a few seconds, substantially increasing efficacy and clinical applicability.The detection of irAEs, especially at an early or subclinical stage, may support timely endocrine testing, closer monitoring of at-risk patients, and early therapeutic interventions such as hormone replacement therapy, corticosteroids, or treatment pauses before severe toxicity develops. Such measures could reduce morbidity, prevent treatment discontinuation, and support continuation of ICI therapy with improved safety.

This study is subject to limitations. First, limited thyroid function tests may underestimate subclinical cases. Moreover, SUV_max_ and SUV_mean_ indicators may be influenced by physiological variability or unrelated treatment effects. Minor segmentation errors could affect quantitative analysis. Further, the number of patients with thyroid-related adverse events was low. However, we found consistent and significant associations across both SUV_mean_ and SUV_max_. In patients with hypothyroidism, uptake increased significantly post-treatment in all cases, supporting a robust relationship between thyroid [^18^F]FDG uptake and irAEs. Nevertheless, effect sizes may be smaller for non-thyroid-related irAEs, and larger cohorts are needed to confirm whether [^18^F]FDG uptake beyond the thyroid is not associated with irAEs. Lastly, AI-assisted opportunistic screening does not replace conventional clinical evaluation and future prospective studies across multiple centers are required to assess the robustness and generalizability of our findings as well as the integration of AI-based quantification tools into routine imaging workflows.

## Conclusion

AI-assisted [^18^F]FDG PET/CT analysis provides a promising avenue for the opportunistic detection of immune checkpoint inhibitor-related adverse events, particularly thyroid dysfunction. Our findings highlight the potential of AI-driven metabolic quantification as an early biomarker for irAEs, which could improve patient management and facilitate timely intervention. While our results underscore the feasibility of this approach, further prospective studies are warranted to refine and validate AI-based PET/CT screening for irAEs across multiple organ systems. Integrating AI into clinical workflows may enhance the diagnostic capabilities of PET/CT beyond conventional applications, paving the way for more personalized and proactive oncologic care.

## Supplementary Information

Below is the link to the electronic supplementary material.

Supplementary Material 1

## Data Availability

Data is available from the corresponding author upon reasonable request.

## References

[1] Ribas A, Wolchok JD. Cancer immunotherapy using checkpoint Blockade. Science. 2018;359:1350–5. 29567705 10.1126/science.aar4060 PMC7391259

[2] Martins F, Sofiya L, Sykiotis GP, Lamine F, Maillard M, Fraga M, et al. Adverse effects of immune-checkpoint inhibitors: epidemiology, management and surveillance. Nat Rev Clin Oncol. 2019;16:563–80. 31092901 10.1038/s41571-019-0218-0

[3] Schneider BJ, Naidoo J, Santomasso BD, Lacchetti C, Adkins S, Anadkat M, et al. Management of immune-related adverse events in patients treated with immune checkpoint inhibitor therapy: ASCO guideline update. J Clin Oncol. 2021;39:4073–126. 34724392 10.1200/JCO.21.01440

[4] Tawakol A, Ishai A, Takx RA, Figueroa AL, Ali A, Kaiser Y, et al. Relation between resting amygdalar activity and cardiovascular events: a longitudinal and cohort study. Lancet. 2017;389:834–45. 28088338 10.1016/S0140-6736(16)31714-7 PMC7864285

[5] Abikhzer G, Treglia G, Pelletier-Galarneau M, Buscombe J, Chiti A, Dibble EH, et al. EANM/SNMMI guideline/procedure standard for [18F]FDG hybrid PET use in infection and inflammation in adults v2.0. Eur J Nucl Med Mol Imaging. 2025;52:510–38. 39387894 10.1007/s00259-024-06915-3 PMC11732780

[6] Cherk MH, Nadebaum DP, Barber TW, Beech P, Haydon A, Yap KS. 18 F-FDG PET/CT features of immune-related adverse events and pitfalls following immunotherapy. J Med Imaging Radiat Oncol. 2022;66:483–94. 35191204 10.1111/1754-9485.13390 PMC9303622

[7] Schierz J-H, Sarikaya I, Wollina U, Unger L, Sarikaya A. Immune checkpoint inhibitor-related adverse effects and 18F-FDG PET/CT findings. J Nucl Med Technol. 2021;49:324–9. 34330805 10.2967/jnmt.121.262151

[8] Muir CA, Clifton-Bligh RJ, Long GV, Scolyer RA, Lo SN, Carlino MS, et al. Thyroid immune-related adverse events following immune checkpoint inhibitor treatment. J Clin Endocrinol Metab. 2021;106:e3704–13. 33878162 10.1210/clinem/dgab263

[9] Stassi G, De Maria R. Autoimmune thyroid disease: new models of cell death in autoimmunity. Nat Rev Immunol. 2002;2:195–204. 11913070 10.1038/nri750

[10] Lokre O, Perk TG, Weisman AJ, Govindan RM, Chen S, Chen M, et al. Quantitative evaluation of lesion response heterogeneity for superior prognostication of clinical outcome. Eur J Nucl Med Mol Imaging. 2024;51:3505–17. 38819668 10.1007/s00259-024-06764-0 PMC11445285

[11] Einspieler H, Kluge K, Haberl D, Schatz K, Nics L, Schmitl S et al. Assessment of PSMA Expression of Healthy Organs in Different Stages of Prostate Cancer Using [68Ga]Ga-PSMA-11-PET Examinations. Cancers [Internet]. 2024;16. Available from: 10.3390/cancers16081514 10.3390/cancers16081514 PMC11049240 38672596

[12] Calabretta R, Hoeller C, Pichler V, Mitterhauser M, Karanikas G, Haug A, et al. Immune checkpoint inhibitor therapy induces inflammatory activity in large arteries. Circulation. 2020;142:2396–8. 32894978 10.1161/CIRCULATIONAHA.120.048708

[13] Calabretta R, Beer L, Prosch H, Kifjak D, Zisser L, Binder P et al. Induction of arterial inflammation by immune checkpoint inhibitor therapy in lung cancer patients as measured by 2-[18F]FDG positron emission tomography/computed tomography depends on pre-existing vascular inflammation. Life (Basel) [Internet]. 2024;14. Available from: 10.3390/life14010146 10.3390/life14010146 PMC10817655 38276275

[14] Calabretta R, Staber PB, Kornauth C, Lu X, Binder P, Pichler V, et al. Immune checkpoint inhibitor therapy induces inflammatory activity in the large arteries of lymphoma patients under 50 years of age. Biology (Basel). 2021;10:1206. 34827199 10.3390/biology10111206 PMC8615286

[15] Wasserthal J, Breit H-C, Meyer MT, Pradella M, Hinck D, Sauter AW, et al. TotalSegmentator: robust segmentation of 104 anatomic structures in CT images. Radiol Artif Intell. 2023;5:e230024. 37795137 10.1148/ryai.230024 PMC10546353

[16] Hinck D, Segeroth M, Miazza J, Berdajs D, Bremerich J, Wasserthal J, et al. Automatic segmentation of cardiovascular structures on chest CT data sets: an update of the totalsegmentator. Eur J Radiol. 2025;185:112006. 39983596 10.1016/j.ejrad.2025.112006

[17] Isensee F, Jaeger PF, Kohl SAA, Petersen J, Maier-Hein KH. nnU-Net: a self-configuring method for deep learning-based biomedical image segmentation. Nat Methods. 2021;18:203–11. 33288961 10.1038/s41592-020-01008-z

[18] Gatidis S, Früh M, Fabritius MP, Gu S, Nikolaou K, Fougère CL, et al. Results from the autopet challenge on fully automated lesion segmentation in oncologic PET/CT imaging. Nat Mach Intell. 2024;6:1396–405.

[19] Virtanen P, Gommers R, Oliphant TE, Haberland M, Reddy T, Cournapeau D, et al. SciPy 1.0: fundamental algorithms for scientific computing in Python. Nat Methods. 2020;17:261–72. 32015543 10.1038/s41592-019-0686-2 PMC7056644

[20] Carlino MS, Larkin J, Long GV. Immune checkpoint inhibitors in melanoma. Lancet. 2021;398:1002–14. 34509219 10.1016/S0140-6736(21)01206-X

[21] Brown LJ, da Silva IP, Moujaber T, Gao B, Hui R, Gurney H, et al. Five-year survival and clinical correlates among patients with advanced non-small cell lung cancer, melanoma and renal cell carcinoma treated with immune check-point inhibitors in Australian tertiary oncology centres. Cancer Med. 2023;12:6788–801. 36404632 10.1002/cam4.5468 PMC10067054

[22] Widakowich C, de Castro G Jr, de Azambuja E, Dinh P, Awada A. Review: side effects of approved molecular targeted therapies in solid cancers. Oncologist. 2007;12:1443–55. 18165622 10.1634/theoncologist.12-12-1443

[23] Wang DY, Salem J-E, Cohen JV, Chandra S, Menzer C, Ye F, et al. Fatal toxic effects associated with immune checkpoint inhibitors: A systematic review and meta-analysis. JAMA Oncol. 2018;4:1721–8. 30242316 10.1001/jamaoncol.2018.3923 PMC6440712

[24] Wright JJ, Powers AC, Johnson DB. Endocrine toxicities of immune checkpoint inhibitors. Nat Rev Endocrinol. 2021;17:389–99. 33875857 10.1038/s41574-021-00484-3 PMC8769055

[25] Iyer PC, Cabanillas ME, Waguespack SG, Hu MI, Thosani S, Lavis VR, et al. Immune-related thyroiditis with immune checkpoint inhibitors. Thyroid. 2018;28:1243–51. 30132401 10.1089/thy.2018.0116 PMC6157359

[26] Aide N, Hicks RJ, Le Tourneau C, Lheureux S, Fanti S, Lopci E. FDG PET/CT for assessing tumour response to immunotherapy: Report on the EANM symposium on immune modulation and recent review of the literature. Eur J Nucl Med Mol Imaging. 2019;46(1):238–50. 10.1007/s00259-018-4171-4 PMC6267687 30291373

[27] Pickhardt PJ, Summers RM, Garrett JW, Krishnaraj A, Agarwal S, Dreyer KJ, et al. Opportunistic screening: radiology scientific expert panel. Radiology. 2023;307:e222044. 37219444 10.1148/radiol.222044 PMC10315516

[28] Sciagrà R. Using artificial intelligence to switch from accident to Sagacity in the serendipitous detection of uncommon diseases. Lancet Digit Health. 2025;7:e104–5. 39890238 10.1016/j.landig.2024.12.006

