# Supplementary material for: Artificial intelligence-enabled opportunistic identification of immune checkpoint inhibitor-related adverse events using [18F]FDG PET/CT
Source: Eur J Nucl Med Mol Imaging. 2025 May 29;52(13):4963–71. doi: 10.1007/s00259-025-07364-2 (PMC12589324; doi:10.1007/s00259-025-07364-2)
Supplement: Supplementary file 1 — Supplementary Material 1 [file 259_2025_7364_MOESM1_ESM.docx]

**Supplemental material**

**Artificial intelligence-enabled opportunistic identification of immune checkpoint inhibitor-related adverse events using [^18^F]FDG PET/CT**

Clemens P. Spielvogel^1^, Aleksa Lazarevic^1^, Lucia Zisser^1,2^, David Haberl^1^, Christophoros Eseroglou^1^, Lucian Beer^3^, Marcus Hacker^1^, Raffaella Calabretta^1^

^1^Division of Nuclear Medicine, Department of Biomedical Imaging and Image-guided Therapy, Medical University of Vienna, Austria

^3^Division of General and Pediatric Radiology, Department of Biomedical Imaging and Image-Guided Therapy, Medical University of Vienna, Austria.

**Correspondence to:**
Raffaella Calabretta, MD
Division of Nuclear Medicine, Department of Biomedical Imaging and Image-guided Therapy

Medical University of Vienna,
Spitalgasse 23
A-1090 Vienna/ Austria
Tel. +43 1 40400 58722

Email: [raffaella.calabretta@meduniwien.ac.at](mailto:raffaella.calabretta@meduniwien.ac.at)

**Definition of thyroid-related adverse events**

Thyroid-related adverse events included hypothyroidism, hyperthyroidism, and autoimmune thyroiditis. Included irAEs were defined as clinically diagnosed inflammatory reaction to the ICI treatment.

In detail, hypothyroidism was defined as TSH levels above the upper reference limit with concomitant fT4 levels below the lower reference interval or elevated TSH levels irrespective of fT4. Hyperthyroidism was defined as suppressed serum TSH levels below the laboratory reference range, in combination with elevated fT4 and/or fT3 concentrations, consistent with increased thyroid hormone activity. Autoimmune thyroiditis was defined as the presence of anti-thyroid peroxidase antibodies and/or anti-thyroglobulin antibodies exceeding the laboratory reference range, and/or a hypoechogenic, heterogeneous thyroid ultrasound pattern indicative of chronic autoimmune thyroiditis, in combination with elevated serum TSH levels.


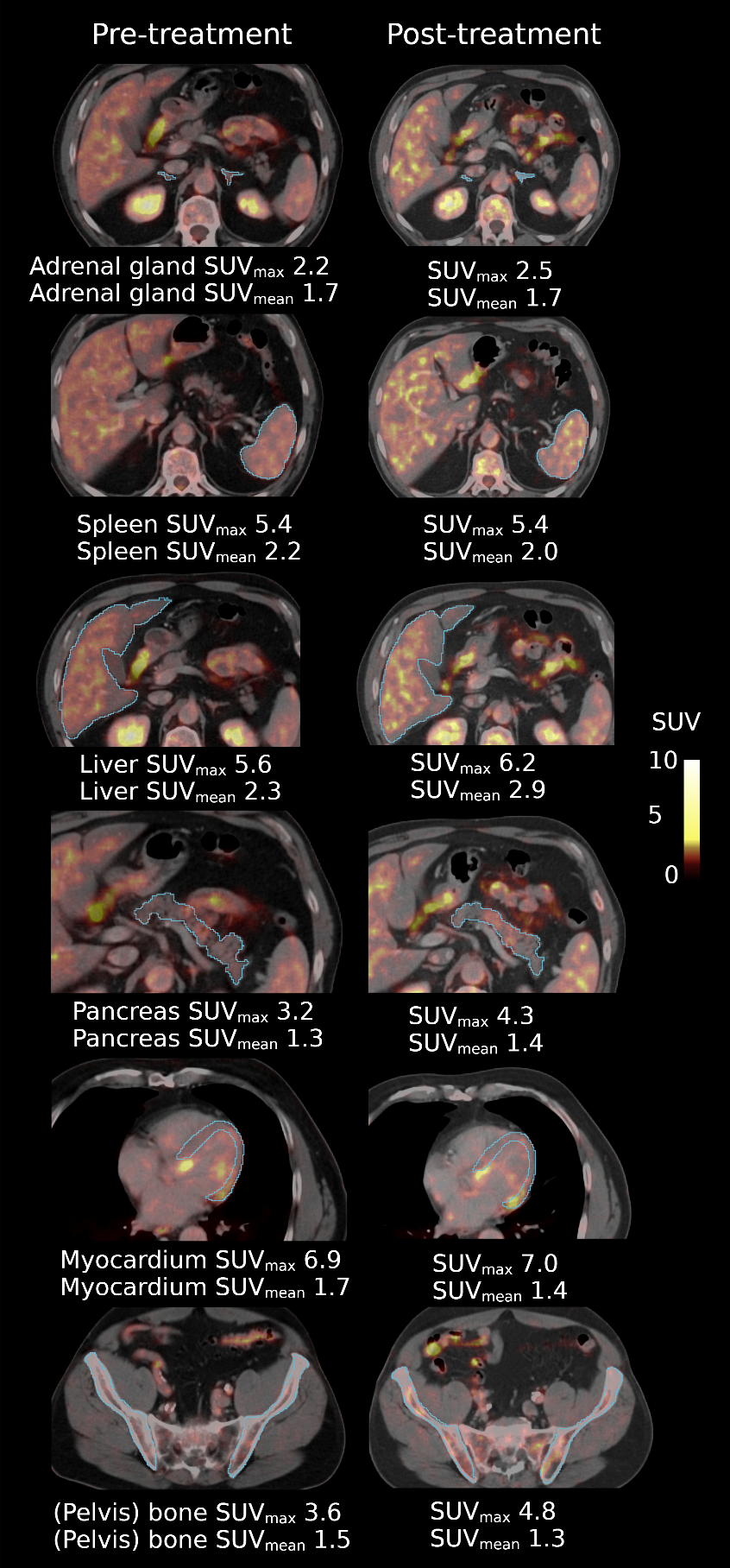


**Supplemental Figure 1.** Transversal slices of an example patient before (left) and after (right) treatment. AI-based delineations are shown outlined in light blue.


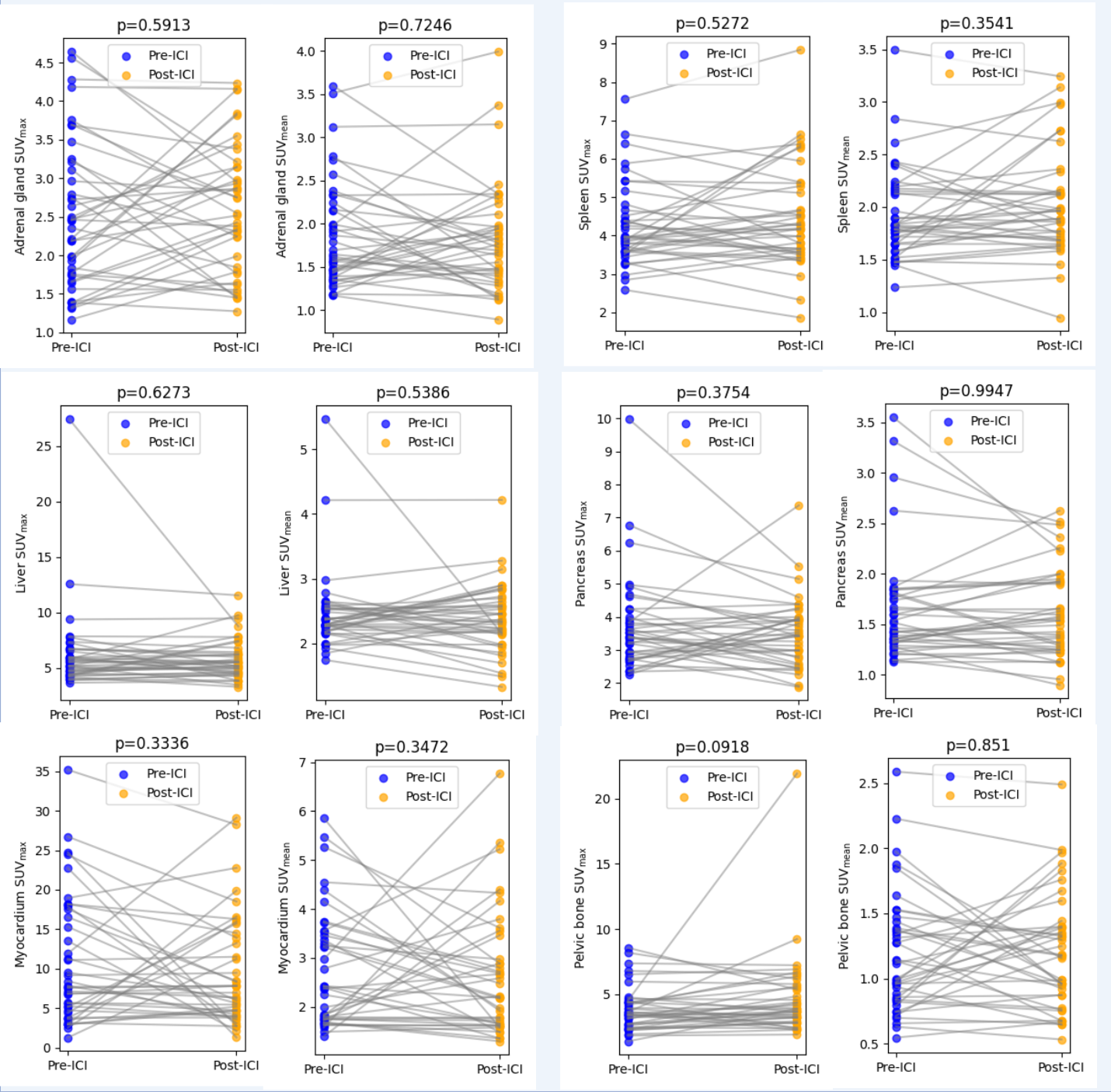


**Supplemental Figure 2.** SUV_max_ and SUV_mean_ for the investigated organs apart from the thyroid before and after treatment.


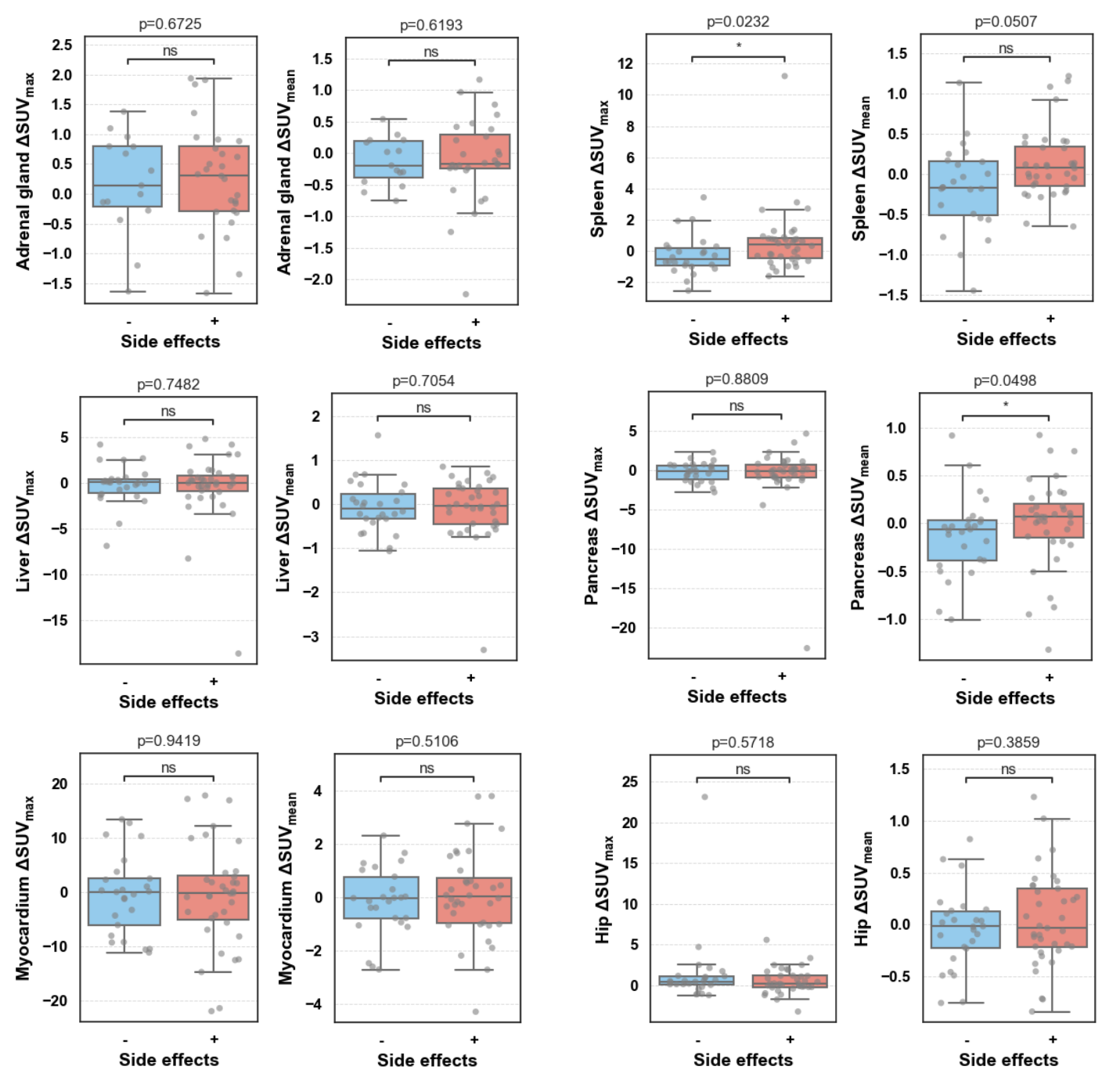


**Supplemental Figure 3.** Changes in SUV_max_ (a) and SUV_mean_ (b) over target organs apart from the thyroid of patients with (+) and without (-) irAEs (side effects).
